# Meckel's Diverticulum Perforation by Foreign Body: A Case Report

**DOI:** 10.30476/BEAT.2021.86253

**Published:** 2021-04

**Authors:** José Roberto Alves, Gustavo Busch Justino, Leonardo Busch Justino, Caique Martins Pereira Ternes, João Vítor Ternes Rech, Fabrissio Portelinha Graffunder

**Affiliations:** 1 *Diseases of the Abdominal Wall and Digestive System Research Group of the Federal University of Santa Catarina, Florianópolis, SC, Brazil*; 2 *Department of Surgery, Polydoro Ernani de São Thiago University Hospital, Federal University of Santa Catarina, Florianópolis, SC, Brazil*

**Keywords:** Meckel's diverticulum, Perforation, Acute abdomen, Emergency medicine

## Abstract

Meckel’s diverticulum is the most common gastrointestinal congenital defect, which, although asymptomatic in adults, may present symptoms in obstruction, inflammation, bleeding and foreign body perforation. There are only 8 reported cases of Meckel’s diverticulum perforation by chicken bone. We report a case of a 24-year-old man presenting a 2-day-history of periumbilical pain that shifted to the right lower quadrant in 24 hours. Clinical and laboratory findings led to an appendicitis diagnosis, followed by laparotomy. Normal appendix was found intraoperatively along with an incidental finding of an inflamed and perforated Meckel’s diverticulum by chicken bone. Diverticulectomy and enteroanastomosis were performed and the patient had a successful recovery, being discharged after 5 days. Although rare, its clinical presentation might be similar to acute appendicitis, which restate the importance of collecting a detailed clinical history and examining the small bowel in order to investigate a possible Meckel’s diverticulum complication in the differential diagnosis.

## Introduction

Meckel’s diverticulum (MD) occurs in 2.2% of the population, representing the most common gastrointestinal congenital anomaly [1, 2]. It is more symptomatic in men, even though the prevalence is similar in male and female patients [1]. MD is a true, short diverticulum with a wide base, composed by the three intestinal layers and it results from a failure in the obliteration and absorption of the vitelline duct, which usually occurs between weeks six and eight of gestation [1, 2]. It is situated in the antimesenteric side of the small intestine, normally 40-100 cm from the ileocecal valve [3]. Its lumen usually contains gastric (60-85%) or pancreatic (5-16%) heterotopic mucosa [1, 3, 4]. 

Less than 2% of patients with MD experience right lower quadrant pain in the first 2 years of life [4] and, afterwards, they become asymptomatic. However, throughout life, complications may arise in 4-40% of cases [5]. Obstruction, inflammation and hemorrhage are the most frequent complications [1]. Cases of perforation are rarer and occur secondary to gangrene, inflammation, intestinal obstruction, peptic ulcer or foreign body ingestion (which represents only 5% of these complications) [5, 6].

We report a rare case of MD perforation due to ingestion of a chicken bone by a young patient that had an appendicitis-mimicking presentation. Only 8 other cases of MD perforation by chicken bone have been described in the current medical literature [1, 4, 7-12].

## Case Report

A 24-year-old white man with mild obesity (BMI=33 Kg/m²) presented to the Polydoro Ernani de São Thiago University Hospital Emergency Department with periumbilical pain for 2 days, that eventually localized to the right lower quadrant in the last 24 hours, associated with nausea and 38°C (100.4°F) measured fever. On the first day of clinical history, he reported having evacuated loose stools on 2 occasions with no signs of blood or mucus. On physical examination, he was in good general condition, hydrated and ruddy, with stable vital signs and with no neurological changes. His abdomen was flat, with global airborne sounds, and tender on palpation in the right flank and lower right quadrant regions, along with a positive Blumberg sign. Laboratory tests showed leukocytosis (17,470 leukocytes/mm³ without left shift) and elevated C-reactive protein. Abdominal and chest radiographs did not show any noteworthy changes.

**Fig. 1:**
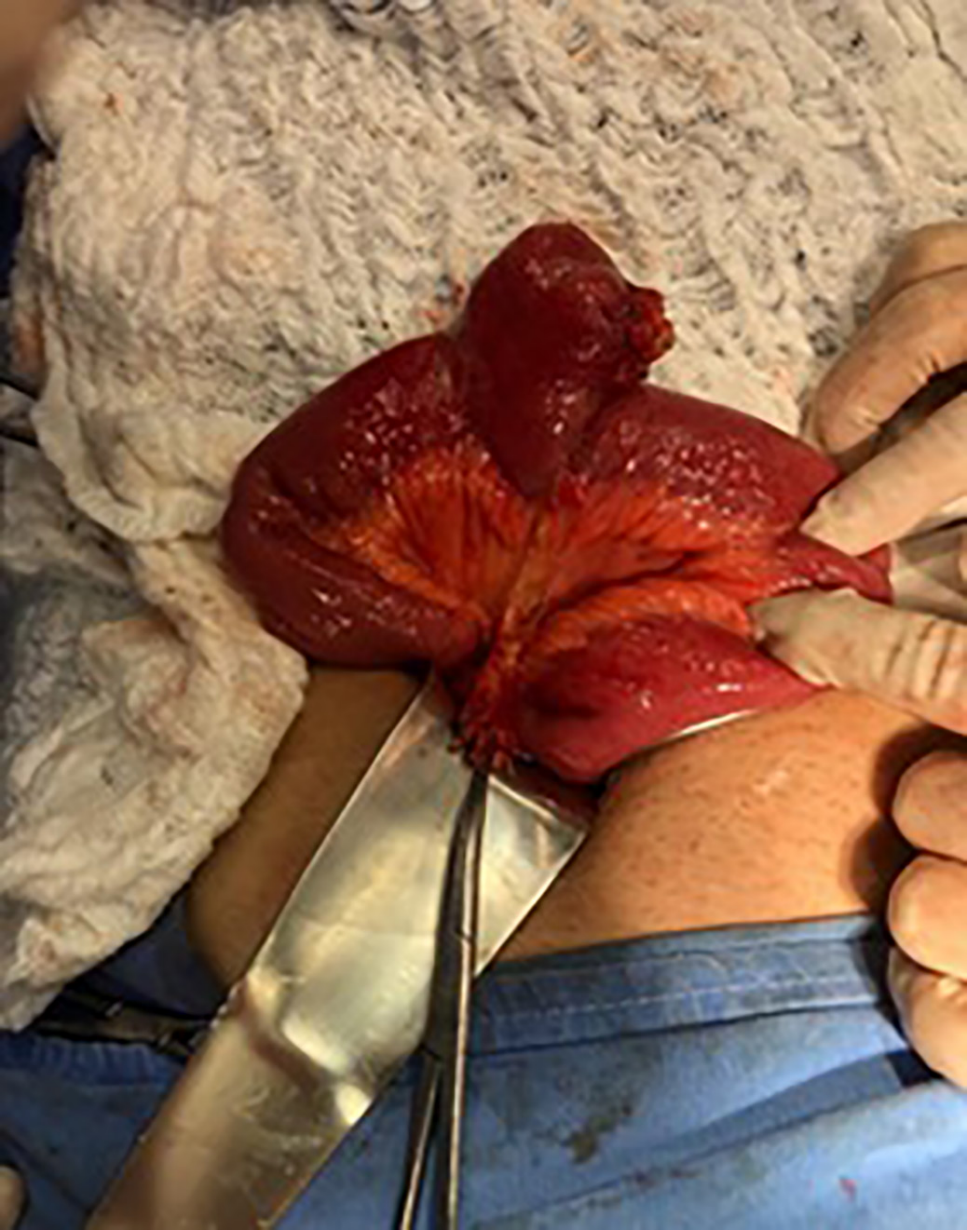
MD on the antimesenteric border, 40 cm from the ileocecal valve

**Fig. 2:**
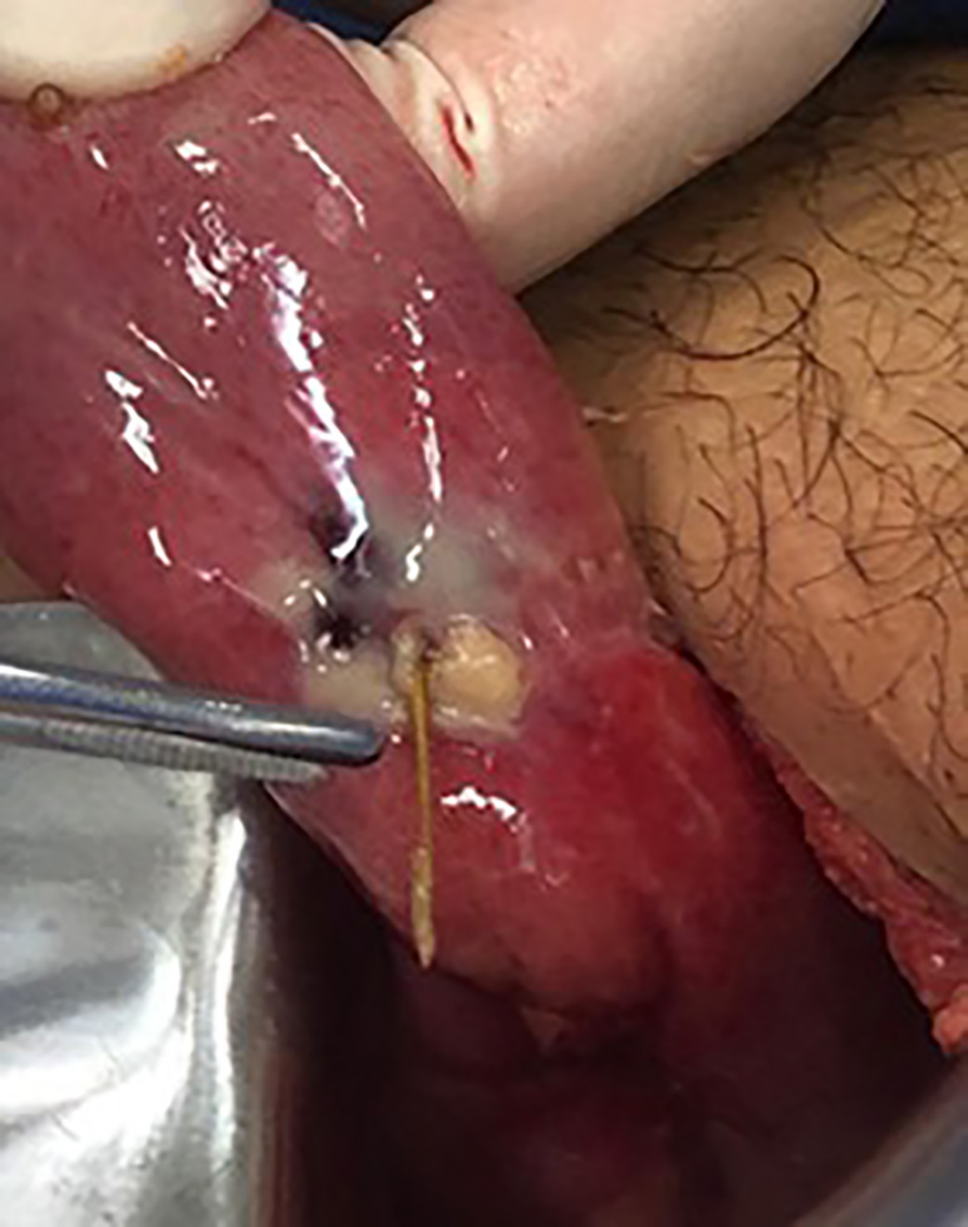
MD wall perforated by chicken bone

Due to strong acute appendicitis clinical suspicion, reinforced by laboratory changes, laparotomy was indicated. McBurney incision was made and the abdominal wall was opened in layers. In the cavity inspection, a retroileal cecal appendix was identified without apparent signs of inflammation. However, a limited amount of purulent liquid was found in the peritoneal cavity. Therefore, a progressive evaluation of the intestinal loops was made and, at a 40 cm distance from the appendix, a perforated MD due to a chicken bone (with an approximate length of 5.5 cm) was identified, associated with fibrin and local phlogosis (Figures 1 and 2).

**Fig. 3:**
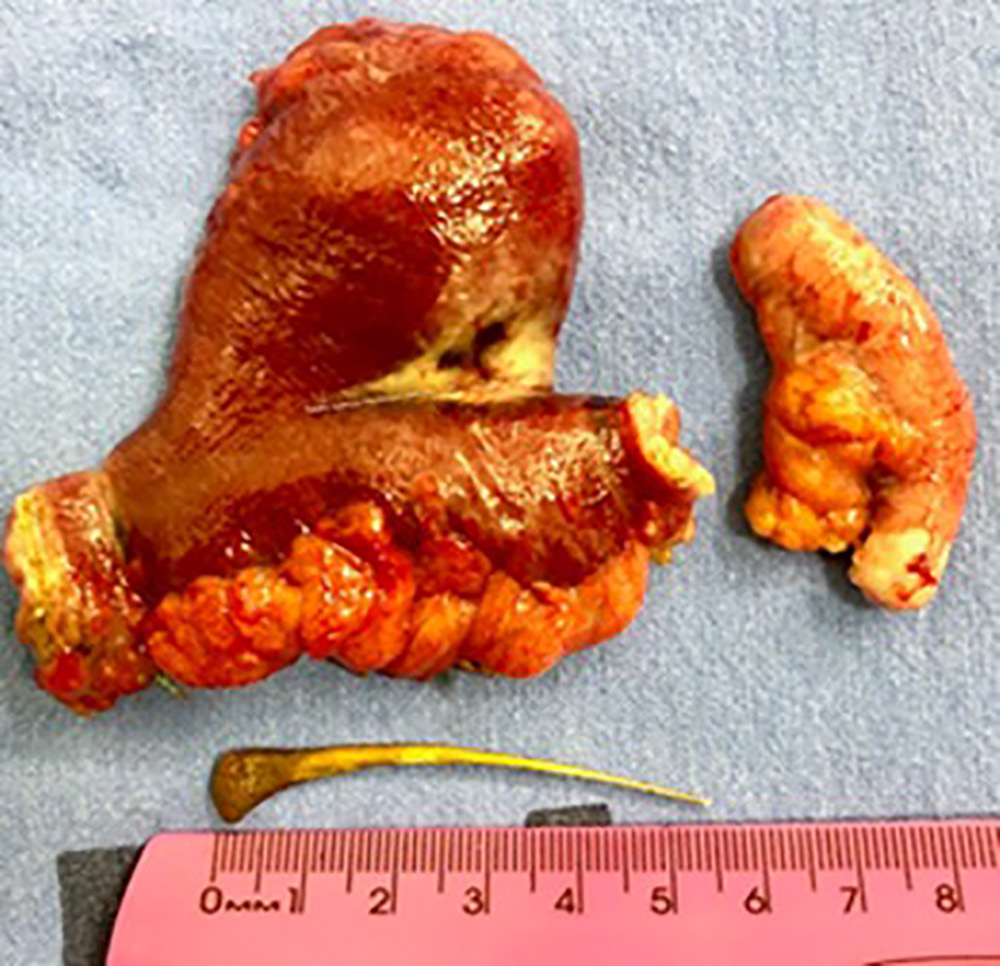
Surgical specimens - enteric segment with resected perforated MD, cecal appendix and 5.5 cm chicken bone

Incidental appendectomy and perforated MD excision with segmental enterectomy (4 cm margin on each side of the diverticulum) (Figure 3) were performed along with posterior end-to-end manual enteroanastomosis, using continuous extramucosal suture with PDS 3.0 thread. Finally, the cavity was cleaned with heated saline and a layered closure of the abdominal wall was performed with non-absorbable threads.

After surgery, the patient was informed about the intraoperative finding (intestinal perforation by chicken bone) and he reported that he had eaten roasted chicken 3 days before the onset of his symptoms, without having initially related to his condition. In the postoperative period, at the hospital ward, he was given antibiotic therapy (ciprofloxacin and metronidazole) and analgesics. Despite initially evolving with postoperative ileus due to lack of ambulation and inappropriate analgesia, the patient accepted oral diet on the 4th postoperative day (PO) and was discharged on the 5th PO after correction of analgesic dosing, constant stimulation for ambulation and administration of antifoaming medication (simethicone). Painkillers and antibiotic supplementation (until 7th PO) were prescribed to be taken at home. Afterwards, he returned to an outpatient consultation on the 14th PO, not referring any complications, with subsequent outpatient discharge after the result of the anatomopathological examination. It confirmed the presence of Meckel’s diverticulum with transmural inflammatory changes in a predominantly acute character, with abscess formation, acute fibrino-leukocytic serositis, free surgical margins and absent heterotopic mucosa after perforation by foreign body. The cecal appendix showed only reactive lymphoid hyperplasia. 

## Discussion

Gastrointestinal tract perforations occur in less than 1% of cases of foreign body ingestion, and only 1.6% of them is localized in the MD [1]. From the complications that can affect MD, foreign body perforation is the rarest [6], and, as in this report, it can mimic acute appendicitis. This might lead to an incorrect diagnosis, considering that patients rarely remember the previous ingestion of a potential foreign body, as it happened in our case [1, 4, 13]. In this case report, the strongly suggestive clinical signs of acute appendicitis (history of periumbilical pain with migration to the right iliac region, associated with low fever and positive Blumberg’s sign) were also found [14]. However, during the intraoperative, no inflammatory process was found in the cecal appendix, and the surgical team correctly proceeded with the expansion of the cavity inspection, that showed a MD perforated by a chicken bone in the small intestine. This is a rare finding, as it is the 9th case described in the medical literature [1, 4, 7, 8-12].

Studies point out the difficulty in diagnosing MD in the preoperative period, due to the absence of symptoms and specific radiological signs [4, 13]. However, a special attention is needed to the differential diagnosis of acute abdomen, especially those that suggest acute appendicitis, particularly in men, due to the possibility of MD's complications (perforation, among others), as described in this case report [1, 15].

Besides performing complementary serum, urinary and preoperative imaging tests (ultrasound and abdominal computed tomography), it is necessary to collect a detailed clinical history in cases of acute abdomen with possible surgical approach. In addition, it is also important to always try to expand the cavity inventory in the intraoperative period, especially in the absence of explanations for the previous clinical findings, with mandatory assessment of, at least, the first 100 cm of the small intestine from the ileocecal valve, in order to find a MD and its possible complications [3, 5, 15].

## Conflict of Interest:

None declared.
